# Hip Fracture Management in a Major Trauma Centre: The Impact of ‘Smart Phrase’ Integration Into Electronic Clerking on Culture and Adherence to Guidelines

**DOI:** 10.7759/cureus.70630

**Published:** 2024-10-01

**Authors:** Benjamin C Barker, Stephen McDonnell

**Affiliations:** 1 School of Clinical Medicine, University of Cambridge, Cambridge, GBR; 2 Trauma and Orthopaedics, Wirral University Teaching Hospital NHS Foundation Trust, Wirral, GBR; 3 Trauma and Orthopaedics, Cambridge University Hospitals NHS Foundation Trust, Cambridge, GBR

**Keywords:** admission proforma, electronic patient record, hemi arthroplasty, neck of femur fractures, nice guidelines

## Abstract

The National Institute for Health and Care Excellence (NICE) introduced guidelines in 2011 for the management of hip fractures in patients over the age of 65. NICE CG124 recommended different procedures depending on the demographics of the patient and fracture pattern.

In terms of compliance in 2019, Addenbrooke’s Hospital (ADH) was found to be in line with the national average, although room for improvement was noted. The aim of this study was to identify and address areas of substantial non-compliance at ADH.

A total of 1636 patients who sustained a hip fracture between 2017 and 2020 and who subsequently underwent surgery at ADH were retrospectively analysed. We collected data from the National Hip Fracture Database (NHFD), digital medical records, and digital imaging systems. We then amended the clerking proforma by adding a ‘smart phrase’ and re-analysed another 543 patients who attended ADH following hip fracture between 2021 and 2022, using the same data collection methods.

From 2017-2020, total adherence to CG124, Section 1.6, was 76.04%. Our results demonstrated that 56.43% of all hip fracture patients indicated for total hip arthroplasty (THA) did not have documented consideration for the procedure. In 2021-22, following the addition of our ‘smart phrase,’ we made to the clerking proforma, total adherence improved to 87.43%, with only 42.11% of THA-indicated patients lacking documented consideration of the procedure, down from 65.41% in 2020. The use of our smart phrase decreased during the follow-up without a corresponding drop in compliance.

In January 2023, NICE CG124 underwent changes, rendering our ADH changes outdated. However, we have demonstrated that we can increase NICE guideline compliance by standardising and encouraging formal documentation of the decision-making process. As electronic patient record (EPR) systems become more widespread, we have shown how ‘smart phrases’ can be used to generate change and increase compliance, even over a short period of time.

## Introduction

Background

The National Institute for Health and Care Excellence (NICE) introduced guidelines in 2011 for the management of hip fractures in patients over the age of 65. The NICE recommendations for surgery are contained within specific national guidelines (NICE CG124) [1], which recommend different procedures depending on the demographics of the patient and fracture pattern. The National Hip Fracture Database (NHFD) [2] recorded that, in 2019, a greater number of hip fracture patients received operative intervention as recommended by NICE following their fracture than in 2018. In terms of compliance, Addenbrooke’s Hospital (ADH) was found to be in line with the national average [3], although room for improvement was noted. Hip fracture care is an important metric when assessing the performance of hospital trusts. NHS trusts are increasingly adopting electronic patient records (EPR) in preference to paper notes; ADH uses Epic, an American-designed EPR.

The aims of this study were principally to assess to what extent NICE CG124 was adhered to when considering surgical management of patients over 65 with hip fractures at Addenbrooke’s Hospital. Specific focus would be given to Section 1.6, which guides the choice of operation based on the type of fracture and other patient factors [1]. Once the compliance has been assessed, we will identify areas where substantial increases in compliance are needed and implement changes to our clerking proforma in the EPR (Epic) to address these. After a sufficient trial period, we would assess again to see if our changes had been associated with an increase in compliance.

## Materials and methods

All patients with hip fractures over the age of 65 who were admitted to Addenbrooke’s Hospital between 2017 and 2020 were retrospectively analysed. This cohort was identified by retrieving data from the NHFD. Detailed data on each patient’s admission was then collected using digital medical records and digital imaging systems. All notes entered on Epic between the time of admission and the time of surgery were used to collect data. Patients who did not undergo surgery for their hip fractures, patients who sustained undisplaced intracapsular neck of femur fractures, and patients who presented with periprosthetic fractures were all excluded from our study, as Section 1.6.3 of NICE CG124 did not apply to these patients. The following data was collected from the records of each patient: (i) Classification of their fracture (based upon imaging reports at the time of admission). (ii) AMTS (Abbreviated Mental Test Score) at the time of admission. Any patient with an AMTS greater than 7 was deemed to fulfil the ‘not cognitively impaired’ criteria set out in CG124. (iii) Mobility status prior to admission (self-reported or from collateral history). (iv) Fitness for surgery (ASA Grade). Any patient with an ASA less than 3 was deemed to fulfil the ‘medically fit for anaesthesia and the procedure’ criteria set out in CG124.

With this information and in concordance with NICE CG124 guidelines at the time (Table 1), we assessed whether the operation type being considered adhered to those stipulated in the national guidelines. Those who had the appropriate surgery, as per the recommendations of the guideline, documented as ‘considered’ were identified and were deemed compliant. Conversely, all patients who did not have the appropriate surgery documented as ‘considered’ were deemed non-compliant. All data were collected using a single observer, who used objective standards of AMTS, mobility, and ASA grade to eliminate intra-observer variations.

**Table 1:** Relevant sections of NICE CG124 (as of June 2021).

Section	Contents
1.6.2	Offer replacement arthroplasty (total hip replacement or hemiarthroplasty) to patients with a displaced intracapsular hip fracture (2017)
1.6.3	Offer total hip replacement rather than hemiarthroplasty to patients with a displaced intracapsular hip fracture who were able to walk independently out of doors with no more than the use of a stick, are not cognitively impaired, and are medically fit for anaesthesia and the procedure (2017)
1.6.7	Use extramedullary implants such as a sliding hip screw in preference to an intramedullary nail in patients with trochanteric fractures above and including the lesser trochanter (AO classification types A1 and A2) (2011)
1.6.8	Use an intramedullary nail to treat patients with subtrochanteric fracture (2011)

After the initial collection of results, Microsoft Excel (Microsoft® Corp., Redmond, WA) was used to calculate levels of compliance expressed as a percentage. Results were also broken down by year. Areas of substantial non-compliance were noted and discussed with members of the trauma team at Addenbrooke’s Hospital. After deliberation, a ‘smart phrase’ was introduced to the clerking proforma (Table 2) in June 2021, which, when used correctly, should have ensured that NICE ‘recommended’ operations are always considered at the first instance.

**Table 2:** A replica of the new smart-text which had been added to the neck of femur clerking proforma at Addenbrooke’s hospital. IC: intracapsular; AMTS: Abbreviated Mental Test Score; THR: total hip replacement.

Intracapsular Neck of Femur Fracture NICE Guidelines	
NICE CG124: Would this patient be suitable for a THR?	
Displaced IC Hip	Yes/No
AMTS > 7	Yes/No
Walks outside with no more than ONE stick	Yes/No
Medically fit for THR	Yes/No
If yes to all – to consider THR	
Discussed with patient	Yes/No
Will discuss and confirm at trauma meeting whether for THR	

Microsoft Excel was used once again to calculate levels of compliance expressed as a percentage. For the data from the second cycle, results were broken down into three-month ‘quarters’. Statistical analysis between the initial cohort and the repeat cohort, comparing both overall compliance and the proportion of eligible patients being considered for THR, was carried out using a two-tailed Z-test performed in Microsoft Excel. Z values were calculated and compared to a table of known values to determine statistical significance.

## Results

Initial cycle

Between 2017 and 2020, 1636 patients sustained a hip fracture and subsequently underwent surgery. The total compliance with NICE CG124, Section 1.6, was 76.04%. Table 3 shows a further breakdown by year.

**Table 3:** Adherence to NICE CG124 at Addenbrookes hospital from 2017-2020. THR: total hip replacement; Hemi: hemiarthroplasty; EM: extramedullary; Nail: intramedullary nail; NICE: National Institute for Health and Care Excellence.

Year	% Appropriate THR considered	% Appropriate Hemi performed	% Appropriate EM implant performed	% Appropriate nail performed	% NICE compliant
2017	40.7	94.6	90.7	54.8	74.4
2018	58	95.3	92	29.6	81.2
2019	43.5	97.3	91.1	47.7	73.9
2020	36.6	91.7	92.5	73.3	75.1
Total	43.6	94.6	91.65	51.7	76

**Figure 1:**
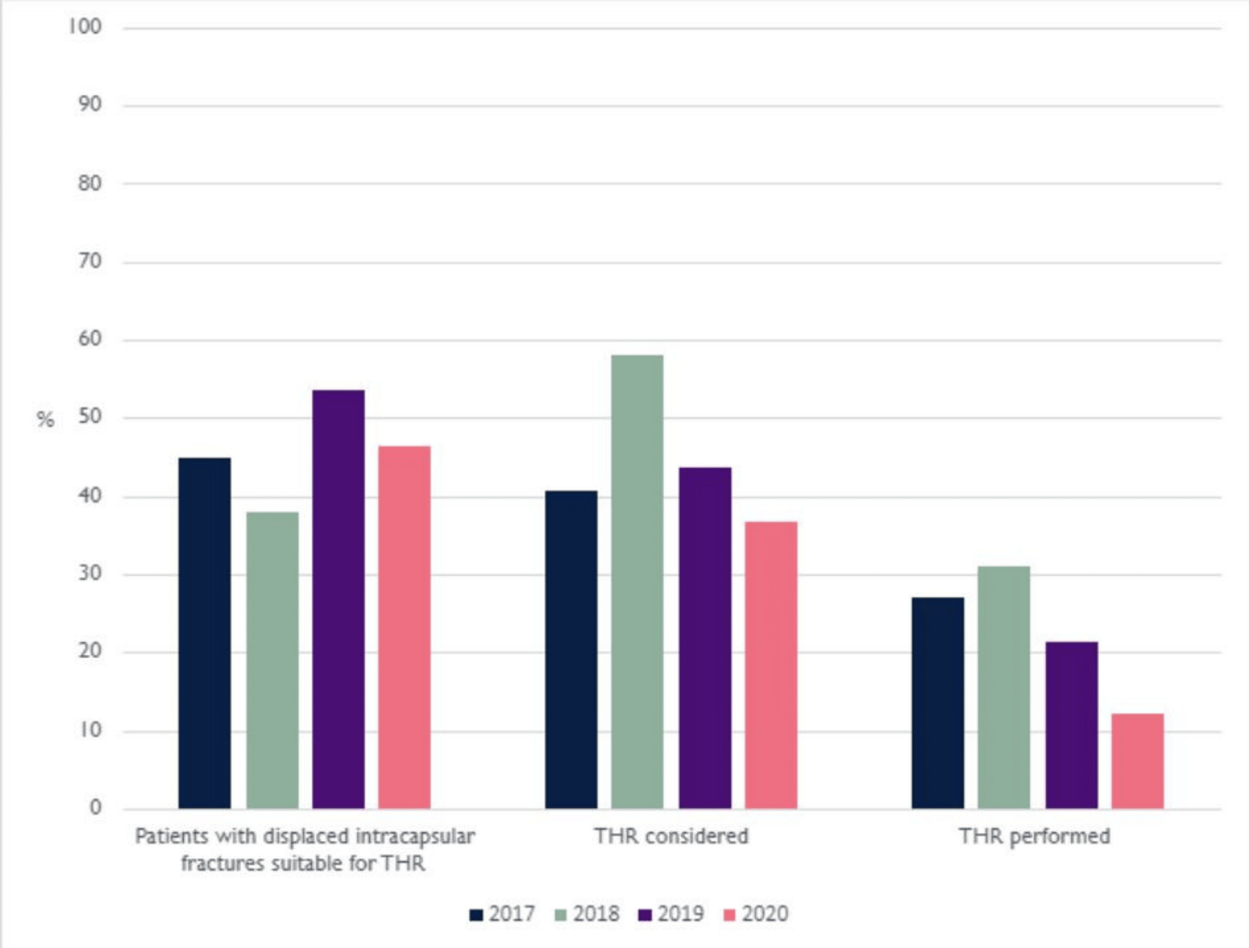
Graph to show the proportion of patients who were eligible for THR, and the proportion of those patients who were considered for, or received THR at Addenbrooke’s hospital from 2017-2020. THR: total hip replacement.

Our results showed that 56.43% of all hip fracture patients who were indicated for total hip replacement did not have documented consideration for the procedure, with the lowest amount recorded in 2020 at 36.59%. Figure 1 shows a breakdown by year.

Second cycle

A total of 612 patients sustained a hip fracture between June 2021 and June 2022. After the same exclusions had been applied as in the first cycle, 543 patients had their records analysed. A summary of the results for compliance, broken down by quarter, is presented in Table 4. The total adherence to CG124, Section 1.6, has significantly improved to 87.43% compared to the 2017-2020 period (z=5.556, p<0.01). Statistical analysis is presented in Table 5. The calculated z-alpha/2 value was compared against the table of known values (Table 6) to produce a p-value of <0.01, indicating that this change was due to chance.

**Table 4:** Adherence to NICE CG124 at Addenbrookes Hospital from June 2021 to July 2022. THR: total hip replacement, Hemi: hemiarthroplasty, EM: extramedullary, Nail: intramedullary nail, NICE: National Institute for Health and Care Excellence. Q1: January, February, March; Q2: April, May, June; Q3: July, August, September; Q4: October, November, December.

Year	% THR considered	% Appropriate Hemi performed	% Apt. EM implant performed	% Appropriate nail performed	% NICE compliant
2021 Jun	64.3	100	88.9	100	83.8
2021 Q3	63	97.8	100	100	89.9
2021 Q4	57.1	97.6	97.7	57.1	84.4
2022 Q1	60.9	100	94.7	100	91.1
2022 Q2	50	100	97.3	100	85.8
Total	57.89	99	96.9	88.9	87.4

**Table 5:** Statistical analysis of overall compliance, compared between the two samples, to calculate a z-value. The change was deemed to be significant (p<0.01) if the value of z-alpha/2 was > 2.576, as per Table 6.

Sample period	Number compliant	Sample size	Proportion compliant	Overall proportion compliant	Calculated z-alpha/2
2017-2020	1244	1636	0.760	0.788	5.556
2021-2022	459	525	0.874		

**Table 6:** Table of known values for the two-tailed Z test used to analyse the results of this study.

P-value	Alpha	Alpha/2	Z alpha/2
0.1	10%	5.0%	1.645
0.05	5%	2.5%	1.960
0.01	1%	0.5%	2.576

**Figure 2:**
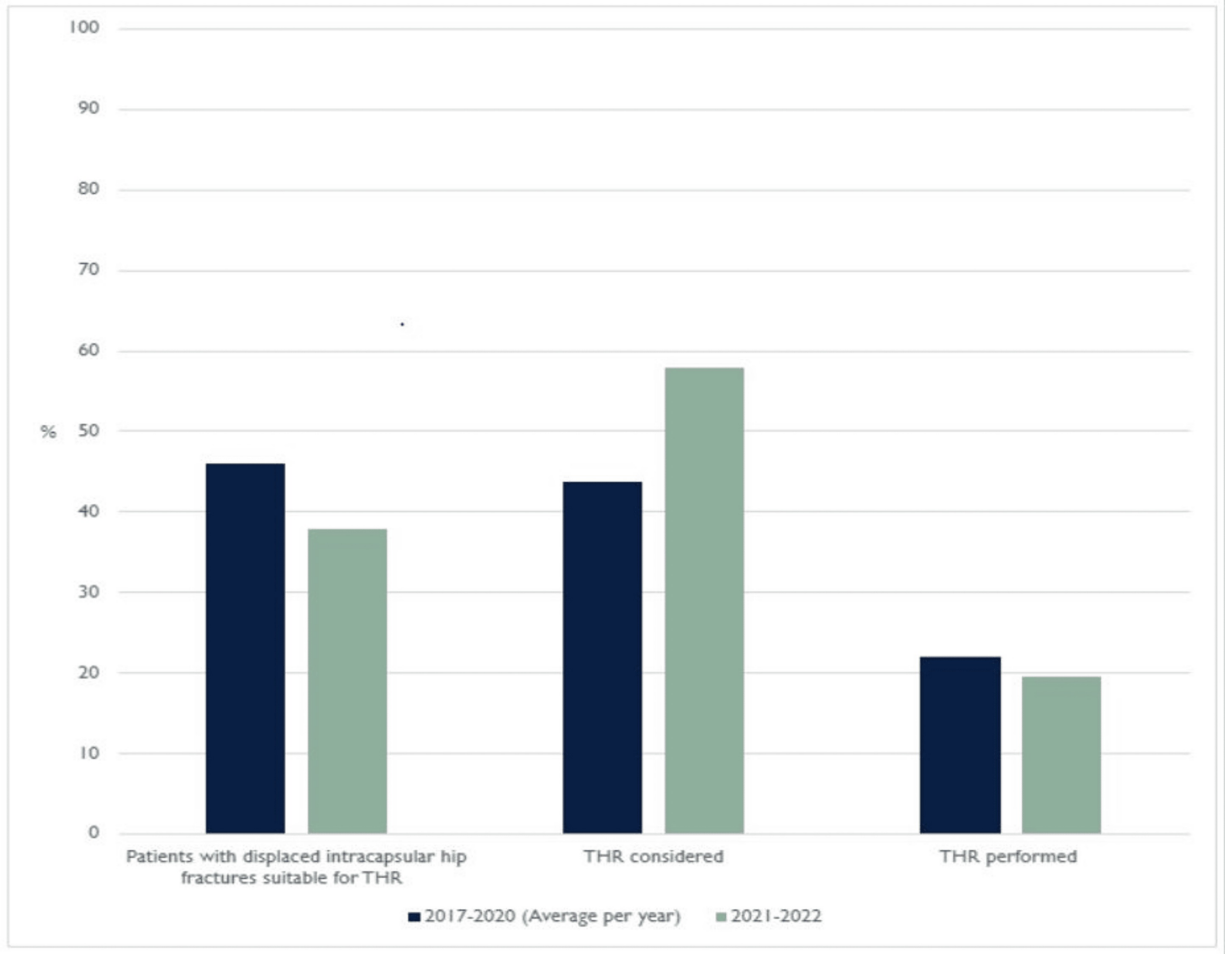
Graph to show the proportion of patients who were eligible for THR, and the proportion of those patients who were considered for, or received THR at Addenbrooke’s hospital in the period from 2017-2020 (yearly average), and the period from June 2021 to June 2022. THR: total hip replacement.

Only 42.11% of patients indicated for THR did not have documented consideration of the procedure, down from 65.41% of patients in 2020. Statistical analysis is shown in Table 7. These results demonstrate a significant increase in documentation of consideration of THR when compared with the 2017-2020 sample (z=2.904, p<0.01). The calculated z-alpha/2 value was compared against the table of known values (Table 6) to produce a p-value of <0.01, that this change was due to chance. Despite this, the percentage of hip fracture patients receiving a total hip replacement did not increase (Figure 2).

**Table 7:** Statistical analysis of documented consideration of THR, compared between the two samples, to calculate a z-value. THR: total hip replacement. The change was deemed to be significant (p<0.01) if the value of z-alpha/2 was > 2.576, as per Table 6.

Sample period	Number documented as THR ‘considered’	Sample size	Proportion documented as THR ‘considered’	Overall proportion documented as THR ‘considered’	Calculated z-alpha/2
2017-2020	193	443	0.436	0.469	2.903
2021-2022	77	133	0.579		

## Discussion

Addenbrooke's Hospital was found to be in keeping with the national average [2] with respect to NICE guidelines for hip fracture operative management. However, the level of care was below the expected standard, and there is scope for improvement. This audit highlighted areas of substantial non-compliance, and we made changes to the clerking proforma with a view to improving NICE guideline compliance. While the management of displaced intracapsular fractures with either total hip replacement or hip hemiarthroplasty in indicated patients remains a matter of debate in the literature [4,5], we felt that we could improve NICE guideline compliance simply by introducing formal documentation of the decision-making process. It is important that, even if hemiarthroplasty is considered to be the preferred option for surgical management, the clinician must make sure the reason for this is clearly documented in the medical notes. We hoped that our smart tool could assist without unduly increasing the workload for the clerking team, who are the first orthopaedic specialists to see the patient once they have arrived in the hospital. In addition to this audit, the data gathered has the potential to be used in order to further research the difference in outcomes between THR and hemiarthroplasty patients.

The results of our re-audit were promising, with increased compliance, particularly in the area that we targeted with our intervention. Anecdotally, use of our proforma decreased over time with clinicians choosing to document their decision process more freely, compliance remaining high through all quarters of the re-audit period.

In January 2023, NICE updated CG124 to change the criteria for considering THR in preference to hemiarthroplasty [1], rendering our ‘smart-phrase’ obsolete. Nevertheless, we feel the change we made had a significant impact, and we envisage that a similar 'smart-phrase'-based approach could be applied to help increase compliance with many other guidelines, regardless of medical specialty. A lot of debate surrounds the phenomenon of ‘checklist fatigue’, but our results do not suggest this is happening within our department. Indeed, previous authors have identified a need for checklists to be concise, adaptable, evolvable, and editable in order to avoid checklist fatigue [6], criteria which we believe our short, editable smart phrase fulfills. A checklist alone cannot change a culture, but in a culture that is receptive to change, even a voluntary checklist has been shown to increase positive outcomes [7]. A 2017 systematic review [8] did suggest that the value of checklists would be increased with accompanying oral prompts but found that electronic checklists were less susceptible to errors than paper checklists. While guideline compliance was found to be increasing, there remained questions over whether this led to direct improvements in patient care. However, as long as guidelines are evidence-based, such as in our example, we would expect this to be the case. A study of changes in an emergency department found that decisions supported by technology led to 83% positive outcomes. However, the authors acknowledge that this field of research currently lacks any randomised controlled trials [9].

The importance of following hip fracture guidelines cannot be understated. A study in Scotland highlighted that sticking to the Scottish standards for hip fracture care did result in improved outcomes for patients [10]. Not only are outcomes better for patients, but when guidelines in hip fracture care are followed, the length of hospital stay is reduced [11], which generates more bed spaces for new admissions. There is a precedent for modifications similar to the one we have implemented; a hospital in Australia managed to increase its department's adherence to local standards by implementing modifications to its EPR as part of a care bundle for hip fractures [12]. 

Outside of orthopaedic surgery, the use of EPR changes to increase patient compliance is widely documented in the literature. Checklists integrated into an electronic proforma have been used to increase compliance in the fields of inflammatory bowel disease [13], cancer care [14], and the management of chronic conditions such as diabetes [15]. An interesting example of EPR changes is presented in the field of ophthalmology, where compliance with more than 30 elements of a guideline was increased following the introduction of changes to the clerking proforma [16]. Interesting to note from this study, however, was that documentation decreased over time, possibly as a consequence of checklist fatigue, as alluded to above. EPR modifications are also particularly useful when, as has happened to NICE CG124, guidelines are continuously modified and updated. During the COVID-19 pandemic, guidance was often being updated, and it was found that the use of EPR could clearly increase the ability of clinicians to keep track of these changes [17].

The use of digital healthcare systems to increase adherence to clinical guidelines is not a new phenomenon, with evidence from the literature recommending its use dating back to the end of the last century [18,19]. Since then, EPRs have become much more widespread and advanced, and the healthcare workforce is likely much more computer-literate. As artificial intelligence (AI) technology becomes ever more accessible and advanced, it is exciting to consider how this technology could be combined with smart phrases such as the one we have used to streamline the process. This potential is already being explored in the field of asthma care [20]. Additionally, the rollout of EPR changes could also be used in resource-constrained communities, and it has been trialed to assist in the management of HIV in the developing world [21].

Limitations 

We are aware this study is limited to one (now outdated) guideline in one centre, and our results do not show the impact of word-of-mouth education of the clinical team based upon the findings of the first phase of our audit. The re-audit period began immediately after implementing our changes, which did not allow us to study trends in compliance over an equivalent time period to the initial sample; as a result, the two samples differ greatly in size. We also note that to ensure consistency in our ‘decision making’ with regard to the choice of surgery, we have used arbitrary cut-off points for both the AMTS and ASA grades, which do not reflect the more subjective process of deciding if a patient is cognitively impaired or fit for surgery, respectively. 

We note that the Epic system used in Addenbrooke’s Hospital has full and easy integration of smart phrases, though this may not be the case for all computer systems.

Additionally, we note that the numbers of THR performed during 2020 may be artificially lower due to trust guidance surrounding the provision of surgery during the height of the COVID-19 pandemic. Nevertheless, this should still have been considered by the orthopaedic team, and the rationale for not choosing to perform the operation should be clearly documented in each case.

## Conclusions

In hip fracture management, we have demonstrated that we can increase NICE compliance by standardising and encouraging formal documentation of the decision-making process. Improvements are possible even with only a short period of implementation of such a change and are likely to be long-lasting, so lengthy as appropriate measures are taken to avoid checklist fatigue. Our change, and changes like it, could be implemented across multiple EPR systems and have applications for several different clinical guidelines. We would recommend the use of smart phrases and electronic clerking proformas for departments that have identified significant areas for improvement when it comes to complying with guidelines.
